# The Role of Donor Leukocyte Infusions in the Treatment of Relapsed Acute Leukemia after Allogeneic Stem Cell Transplantation: A Retrospective Analysis

**Published:** 2018-07-01

**Authors:** Mohammad Vaezi, Mohammad Zokaasadi, Shervin Shahsavari Pour, Amir Kasaeian, Mohsen Nikbakht, Hosein Kamranzadeh Fumani, Kamran Alimoghaddam, Ardeshir Ghavamzadeh

**Affiliations:** Hematology, Oncology and Stem Cell Transplantation Research Center, Tehran University of Medical Sciences, Tehran, Iran

**Keywords:** Leukemia, Hematopoietic stem cell transplantation, Recurrence, Survival analysis, Donor leukocyte infusions

## Abstract

**Background:** Allogeneic hematopoietic stem cell transplantation (allo-HSCT) is the only treatment offered for acute leukemias with potential curative capability. One of the main reasons of treatment failure in patients after allo-HSCT is return of the primary disease. This study aimed to evaluate the role of different modalities available to treat the patients with relapsed acute leukemia after allo-HSCT, focusing mainly on donor leukocyte infusions (DLIs).

**Materials and Methods: **This study included 277 patients who relapsed after myeloablative allo-HSCT between February 2003 and February 2015. Treatment option was offered to all patients, but it was not accepted by about one-third of the study participants. Treated patients were categorized based on receipt of DLI (DLI-based vs. non DLI-based). The effect of treatment in all patients and then the effect of DLI among the treated group was evaluated. Kaplan-Meier method was used for calculating survival rates. All patients were relapsed cases, thus only overall survival (OS) was calculated**.**

**Results: **One hundred and forty-five ALL patients and 132 AML patients were included in the study. One year survival rate for treated patients was 25.13% and for patients who received best supportive care was 2.79% (P<0.001). The difference was significant in both AML and ALL groups. Using DLI-based treatments were accompanied by a noticeably superior outcome. Hazard ratio was 0.43 (0.29-0.63) for DLI-based treatments (P<0.001).

**Conclusion: **Despite the poor prognosis of relapsed acute leukemia after HSCT, it seems that treatment interventions and, especially DLI-based treatments, can be of substantial benefit for patients.

## Introduction

 Acute leukemias are among the most frequent hematological malignancies and until now the only treatment offered with a potential curative capability is allogeneic hematopoietic stem cell transplantation (allo-HSCT) ^1^^,^^2^ . Since the advent of more advanced methods to decrease the transplant- related mortality (TRM) mainly from graft-versus-host disease (GVHD) and life threatening infections, one of the deadliest and most difficult outcomes to manage is the relapse of the primary disease ^3^^,^^4^ . Relapse occurs mostly in the first 1-2 years after transplant and holds the biggest share of late deaths ^5^^,^^6^ . The prognosis of relapse after allo-HSCT is poor and only a few modalities are available to ameliorate the patients’ conditions^        7 ^. These options include aggressive chemotherapy, donor leukocyte infusions (DLIs) and, in some cases, radiotherapy.

Despite promising results in some conditions like chronic myeloid leukemia (CML), DLI was not reported as successful as anticipated in the first experiences for acute leukemia ^8^^-^^10^ . The results were particularly disappointing in acute lymphoblastic leukemia (ALL)  ^11^^,^^12^ , and until present its efficacy, safety and toxicity is not fully established in acute leukemia. This study was designed with the purpose of evaluating the role of treatment, especially DLI, in relapsed acute myeloid leukemia after allo-HSCT at our center.

## MATERIAL AND METHODS

**Patient eligibility, data collection and ethical considerations**

From February 2003 to February 2015, a total number of 277 patients with relapsed acute leukemia (either acute myelogenous leukemia; AML or acute lymphoblastic leukemia; ALL) after allo-HSCT were selected for the study. The study was conducted at the Hematology, Oncology and Stem Cell Transplantation Research Center, Tehran, Iran. Diagnosis of primary disease was made based on clinical condition of the patients, morphologic features of bone marrow aspiration, biopsy and flowcytometry. Relapses were classified as systemic or isolated extramedullary (IEM) (in cases of both involvements, the systemic component was prevailed over). The only exclusion criterion was acute promyelocytic leukemia. Informed consent was obtained from all patients to use their medical records as a material for medical research. The study protocol was approved by Ethics Committee of the Hematology, Oncology and Stem Cell Transplantation Research Center. There is no conflict of interest to disclose.

Variables, including age, sex, primary disease and subtype, times to relapse and death, treatment type(s) after relapse and survival status of the patients were extracted from their medical records. Patients who were lost to follow-up were contacted to update their survival status. Treatment at our center was planned for all patients, but some of them refused to take it. Therefore, patients were divided into two arms: one arm who received only best supportive care (BSC) and another arm who did receive at least one treatment modality. After survival analysis, the first arm was omitted from analysis and the second arm was classified based on the type and number of treatments. Patients who received DLIs (either alone or in combination with other modalities) were categorized as DLI-based group and non-DLI based group (other treated patients). In order to calculate relapse incidences, the starting point was the time of HSCT and for survival analysis was the time of relapse.

**Primary HSCT**

The allo-HSCT procedure from full-matched donors at our center was preceded by a myeloablative conditioning regimen. This regimen consisted of busulfan 0.8 mg/kg intravenous (IV) or 4 mg/kg orally (PO) three times a day (TDS) for 4 days (day -6 to -3), and cyclophosphamide 60mg/kg IV daily for 2 days (-2 and -1). GVHD prophylaxis was prearranged by methotrexate 10 mg/m^2^ on day +1 and 6 mg/m^2^ on days +3, +6 and +11 plus cyclosporine started from day -2 in the following dosages: 1.5 mg/kg/day IV (intravenous) from day -3 to +7, 3mg/kg/day IV from day +8, and then continued until the patient could tolerate oral, then the drug shifted to 6 mg/kg/day PO. If chronic GVHD occurred, the treatment was continued and if no GVHD was present after 3 months treatment was stopped. Folinic acid was administered four times a day (QID) on days 2,4,5,7 and 8 after transplant and two times on day 9. Stem cells were collected from peripheral blood of donors. Body irradiation was not part of the preparation routine.

**Diagnosis of relapse and post-relapse intervention protocols**

Relapses were screened and diagnosed whenever one of the following events occurred: a decrease in mixed chimerism, minimal residual disease (MRD) detection by molecular or flowcytometry studies, alterations in complete blood count (CBC) or bone marrow aspiration and biopsies in favor of disease recurrence. The first step in the management of patients after relapse was salvage chemotherapy which consisted of vincristine 2mg IV weekly plus dexamethasone 8mg IV BD for ALL patients and cytarabine (20 mg Day1-Day5 per week) plus interferon-α (3 million units three times per week) for AML patients. When the patients had IEM relapse, radiotherapy (RT) was added to the chemotherapy and if the patient did not accept the chemotherapy, then RT would remain the only therapeutic modality. Treatment efficacy at this step was evaluated by an increase in chimerism, bone marrow aspiration and biopsy (BMA/B). After decreasing the tumor burden and increasing the donor chimerism, patients received DLIs. No appropriate response to primary salvage protocol led to 3 consecutive days of salvage chemotherapy using daunorubicin. DLI process at our center was non GCSF-primed and started from 1x10^7^ CD3+ cells. Due to cost considerations, we did not positively select CD3+ cells; therefore, the final infused product consisted of a leukocyte infusion which included the same number of counted CD3+ cells. In responder patients, the same amount was continued for 3 cycles and in non-responders the cell counts were escalated to 2x10^7^ cells and then 1x10^8^ cells. The time interval between DLIs was one month. GVHD was controlled at the time of DLIs, but in case of active GVHD after DLIs, they were discontinued. By the end of DLIs, mixed chimerism was rechecked by means of PCR. Maintenance of treatment consisted of cytarabine plus interferon-α for AML patients and vincristine plus dexamethasone for ALL patients.

**Statistical analysis**

 The data were analyzed in retrospect and survival rates were computed based on Kaplan-Meier estimate. All studied patients were relapsed cases, therefore only overall survival (OS) rates were calculated. The median follow-up time was calculated using reverse Kaplan-Meier method. The effects of treatment options were analyzed via cox proportional hazard model and compared by log-rank test. Variables with a P-value of less than 0.2 entered the multivariate analysis. These variables included treatment, site of relapse for all patients and DLI-based interventions for treated patients. Statistical level of significance was defined as P-values of less than 0.05. Analyses were done in R for windows version 3.2.2 and Stata statistical software (version 11.2).

## Results

**Basic results and demographics**

A total number of 277 patients were included in this study. The mean age at transplantation was 29.83±10.30 years (27.18±9.51 for ALL and 32.74±10.38 for AML). One hundred and ninety patients (68.59%) received treatment and the others received BSC (31.41%). Among the treated group, 57 patients (30%) were treated by DLI-based treatments. The basic characteristics of studied cohort are demonstrated in Table 1.

**Table 1:** Basic characteristics of the patients

**Covariate**	**Category**	**Frequency**
Disease type	AML	47.65% (n=132)
	ALL	52.35% (n=145)
Sex	Male	63.90 %( n=177)
	Female	36.10 %( n=100)
Donor type	Full-matched sibling	100% (n=277)
Relapse type	Systemic	79.42% (n=220)
	Isolated extramedullary	20.58% (n=57)
Treatment after relapse	Treatment	68.59% (n=190)
	BSC	31.41% (n=87)
Treatment based on DLI*	DLI based	30% (n=57)
	Non DLI based	70% (n=133)
Number of treatment modalities used*	Single treatment	68.95% (n=131)
	Multi-modality treatments	31.05% (n=59)

* percentages are computed only among treated patients

Systemic relapse was observed in 79.42% of patients (n=220), of whom 40.43% (n=112) had ALL and 38.99% (n=108) had AML. Moreover, extramedullary relapse was seen in 20.58% of patients (n=57), of whom 24 (8.66%) had AML and the remaining 33 (11.92%) had ALL. The site of extramedullary relapses was CNS (n=7), breast (n=5) and other sites (n=12) in AML and CNS (n=15), testis (n=4), breast (n=3) and other sites (n=11) in ALL patients. The other categories in AML patients included musculoskeletal system (n=5), solid organs (n=3), reticuloendothelial system (n=3) and pelvic mass (n=1). The similar classification in ALL patients consisted of musculoskeletal system (n=3), solid organs (n=3), reticuloendothelial system (n=2) and soft tissue involvement (n=3). The median time to relapse for all patients was 181 days from the time of HSCT. On average, systemic relapses occurred earlier (median time to relapse: 170 days for systemic and 297 days for IEM relapses). Also, the median time from HSCT to relapse was higher in ALL patients (190 days vs. 171 days among AML group). Most of the relapses occurred within the first year after allo-HSCT (75.44%).

Ten patients received radiotherapy as the only therapy modality, of whom 9 were in the IEM group and one patient had both systemic and IEM relapses. About one third of systemic relapses were treated with DLI-based treatments (64.5% of systemic group), but this portion in IEM group was considerably lower (15.40%).

In the systemic group, treatments were as follows: 84 patients (60.84%) received chemotherapy alone, 1 patient (0.73%) RT alone, 17 patients (12.33%) DLI, 29 patients (21.02%) chemotherapy plus DLI and the remaining 7 patients (5.08%) received chemotherapy and RT ± DLI.

Among the IEM group, the most frequently used treatment modalities were chemotherapy alone (36.54%) or in combination with radiotherapy (30.77%). Other interventions included radiotherapy alone (17.31%), DLI alone (1.92%), DLI and RT (1.92%) and a triple combination of DLI, chemo and radiotherapy (11.54%).

**Survival analysis**

**Figure 1:**
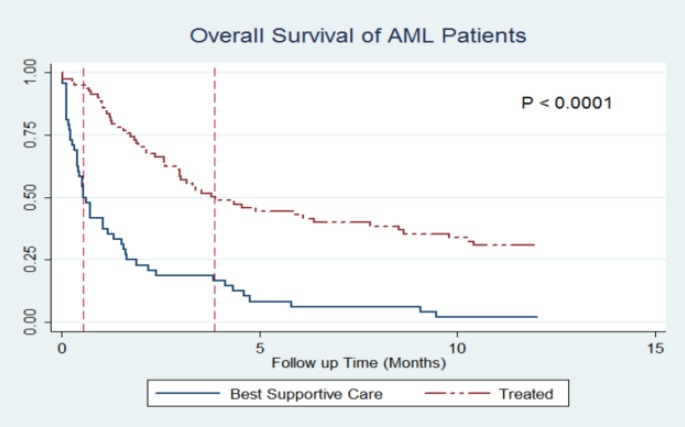
Kaplan-Meier curves of treatment vs. best supportive care in AML patients.

**Figure 2:**
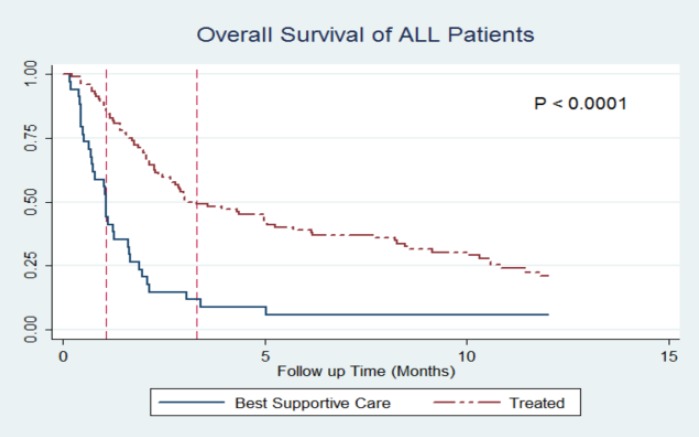
Kaplan-Meier curves of treatment vs. best supportive care in ALL patients.

By the end of the follow-up time, 84.12% of patients (n=233) died. Causes of death for all, but two patients, were disease relapse or its complications. Two patients were suffering from severe GVHD after DLIs and eventually succumbed to it. All patients, but 55 with severe GVHD, in the DLI group received 3 DLI cycles with the same dosage explained in the methods. In a median follow-up of 12 months, Kaplan-Meier estimate revealed 1-year overall survival rate of 17.93% for all patients. With regard to treatment, the probability of survival was significantly higher among the treated group than BSC group (1-year OS: 25.13% vs. 2.79%; P<0.001), and the difference was seen in both AML (1-year OS: 30.79% vs. 2.00%; P<0.001) and ALL (1-year OS: 20.74% vs. 4.86%; P<0.001) patients (Figures 1&2).

The median OS time among patients were 34 vs. 106 (ALL group) and 48 vs. 82 days (AML group) in the BSC and treatment groups, respectively. The probability of survival was also significantly different between the systemic relapsed group and IEM (one-year OS; 17.33% vs. 44.47%; P<0.001). Amongst the patients who received at least one form of treatment, the analysis showed that DLI-based treatments are associated with significant survival advantage compared to non-DLI based treatments (Table2).

**Table 2:** Effect of DLIs on the overall survival in treated patients

**Treatment**	**Category**	**1-year ** **OS**	**3-year ** **OS**	**5-year ** **OS**	**P-value**
DLI	DLI based	40.83%	22.46%	22.46%	<0.001
	Non-DLI based	18.50%	4.03%	0	

**Figure 3:**
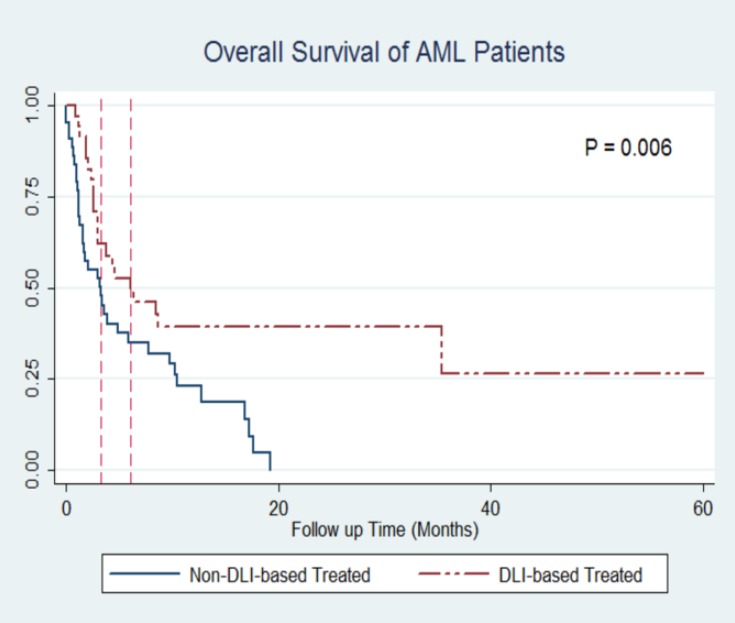
Kaplan-Meier curves of DLI based vs. non-DLI based treatment in AML patients.

**Figure 4:**
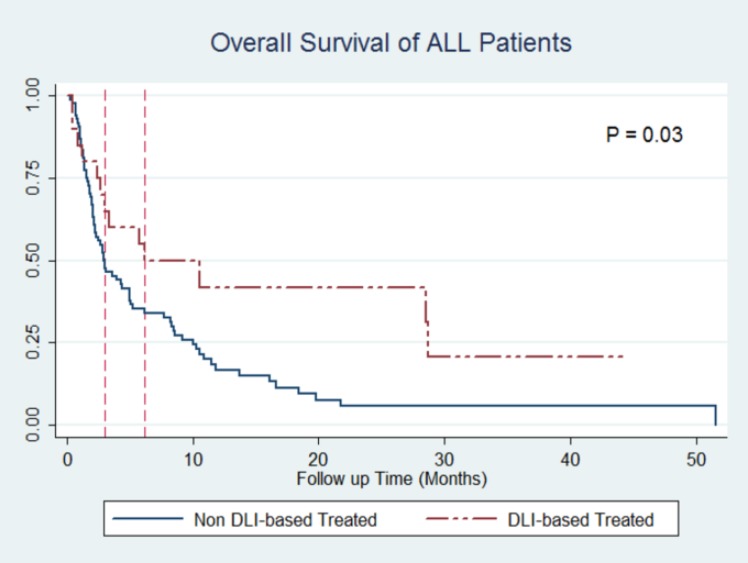
Kaplan-Meier curves of DLI based vs. non-DLI based treatment in ALL patients.

Furthermore, it should be noted that the effect of DLI on outcome was observed in both diseases and was independent of relapse type. The 1-year probability of survival with regard to DLIs for AML patients was 31.93% (95% CI: 15.64% - 49.53%) vs. 8.30% (95% CI: 1.46% - 23.06%), P=0.006, Figure 3. Among ALL patients, the same probabilities were 41.47% (95% CI: 18.64% - 63.05%) vs. 16.37% (95% CI: 9.04% - 25.60%), P=0.03, Figure 4.

Median OS time in DLI and non-DLI-based groups were 86 vs. 20 days in ALL patients and 45 vs. 37 days in AML patients.

**Cox proportional hazards model**

In the univariate and multivariate analyses of all patients, isolated extramedullary relapse and treatment were significantly related to better consequences. Among the treated patients, DLI-based treatments led to superior outcomes.

Results of univariate and multivariate analyses are summarized in Table 3.

**Table 3:** results of univariate and multivariate analysis

**Covariate**	**Univariate**		**Multivariate**	
	**Hazard ratio ** **(95% CI)**	**P-** **value**	**Hazard ratio ** **(95% CI)**	**P-** **value**
age	1.01 (0.99- 1.02)	0.44		
Sex (M/F)	0.97 (0.73- 1.29)	0.82		
Disease type (AML/ALL)	1.07 (0.82- 1.41)	0.61		
Treatment vs. BSC	0.29 (0.22- 0.39)	<0.001	0.32 (0.24- 0.43)	<0.001
Relapse site (IEM/systemic)	0.38 (0.26- 0.57)	<0.001	0.45 (0.30- 0.66)	<0.001
DLI based treatment*	0.48 (0.33- 0.71)	<0.001	0.43 (0.29- 0.63)	<0.001

IEM: isolated extramedullary, CI: confidence interval.

*after dropping “BSC” group from analysis.

DLIs in AML and ALL patients were shown to be independently associated with an improved outcome. The hazard ratio (HR) of DLI-based treatments for all patients was 0.43 (95% CI: 0.29-0.63; P<0.001). Among AML and ALL groups, after adjusting for the same possible confounders, the HR was 0.35 (95% CI: 0.22-0.68; P=0.001) and 0.46 (95% CI: 0.25-0.85; P=0.01), respectively.

## Discussion

 This study was aimed to evaluate the role of treatment, particularly DLI-based treatments, in the management of relapse in acute leukemia after allogeneic HSCT. As cited earlier herein, first experiences of DLI in acute leukemia were not auspicious starts, nevertheless, more recent prospective studies revealed that DLI-based treatments might potentially have a role in the management of relapse after allo-HSCT (13, 14).

In spite of a generally poor prognosis of relapsed acute leukemia after allo-HSCT, it seems that treatment interventions could be advantageous for the patients. In our study, the probability of survival for AML patients at first year was 30.79% (with treatment) vs. 2.00% (with BSC). The same difference was also observed among ALL patients (1-year OS: 20.74% vs. 4.86%). This finding is in line with the literature. The study of Arellano et al. which was a report from one center resulted in similar findings. They found out that the chemotherapy/supportive care alone led to inferior outcomes in comparison to second transplant, DLI or cytokines^        15 ^. Therefore, it could be assumed that even with unsuccessful outcomes of relapse after allo-HSCT, it is better to offer the benefits of treatment modalities to patients.

Also, 1- and 3-year OS rates in the group treated with DLI (either alone or in combination with radio or chemotherapy) were 40.83% and 22.46%, respectively which is in line with the literature. The prospective studies of Choi SJ et al. on 10 ALL and 16 AML patients with DLI preceded by chemotherapy showed 1- and 2-year OS rate of 40% and 20% for ALL patients and 38% and 31% for AML patients, respectively ^13^^,^^14^ . Larger studies like the multicenter report of Collins RH et al. on 44 ALL patients showed 13% OS rate after 3 years^        16 ^. In their report, 28 patients were treated with chemotherapy followed by DLI. Aside from chemotherapy before DLI and number of DLIs, only a small fraction of patients (3 out of 44) have had long-lasting remissions of one year or more.

We use myeloablative conditioning regimen (Bu/Cy) prior to allo-HSCT for acute leukemia. It should be noted that studies with non myeloablative preparations have not reached to satisfactory outcomes as well^   17 ^.

Overall, majority of former studies were not capable of revealing a favorable outcome following intensive chemotherapy in the treatment of relapsed acute leukemia, but the modalities focusing on augmentation of immune responses against malignant cells such as DLI, which supposedly could lead to a stronger graft versus leukemia (GVL) effect and superior outcomes went along with more consideration^        15 ^.

A number of existing reports revealed that the decline in donor chimerism of bone marrow B cells, T cells and natural killer cells and also CD34+ peripheral blood cells could herald a hematologic relapse and act as indicator for detection of minimal residual disease (MRD) ^18^^-^^20^ .

The retrospective nature of this study, in addition to non-randomized distribution of population in different groups (mainly between treated and non-treated groups), could be a potential hindrance in order to attain a clear and sturdy interpretation of the findings although the multivariate analysis model could adjust and compensate the effect of potential confounders. At our center, we do not perform second transplant which is offered to be efficacious in some studies^        21 ^. The mechanism of action presented for second transplant is to some extent the same as DLI (stimulating the GVL effect), chiefly in cases of changing the donor to an unrelated match. The latter approach has recently came to application^        22 ^.

## CONCLUSION

 In general, DLI for relapsed acute leukemia is still not working as a potent immunotherapeutic ideal and is not capable of re-induction and maintenance of long- lasting remissions in an acceptable percentage of patients. This could be to some extent due to an imbalance between slow onset of acting for GVL effect of DLIs, the rapid growth of malignant cells in acute leukemias^17^ and also the energy of T cells induced by cancer cells that has been shown in some experimental models^        23 ^. Novel techniques such as engineered cytokine-induced killer cells (CIKs)^        24 ^ as well as neat strategies to screen the molecular relapse before the presence of an overt hematological relapse are needed to be developed to enhance the efficacy of the procedures and subsequently survival of the patients. Also, developing and usage of modified therapeutic DLIs might lead to better outcomes   ^25^^, ^^26^. But, until then, it seems that the necessity of treatment and, especially GVL enhancing modalities like DLI and their survival benefits, should be considered as one of the best existing approaches for an inevitably, extremely fatal disease.
